# Diffusion Tensor Magnetic Resonance Imaging of the Pancreas

**DOI:** 10.1371/journal.pone.0115783

**Published:** 2014-12-30

**Authors:** Noam Nissan, Talia Golan, Edna Furman-Haran, Sara Apter, Yael Inbar, Arie Ariche, Barak Bar-Zakay, Yuri Goldes, Michael Schvimer, Dov Grobgeld, Hadassa Degani

**Affiliations:** 1 Department of Biological Regulation, Weizmann Institute of Science, Rehovot, Israel; 2 Sackler School of Medicine, Tel-Aviv University, Tel-Aviv, Israel; 3 Institute of Oncology, Sheba Medical Center, Tel Hashomer, Israel; 4 Unit of Biological Services, Weizmann Institute of Science, Rehovot, Israel; 5 Division of Diagnostic Imaging, Sheba Medical Center, Tel Hashomer, Israel; 6 Department of Hepato-Pancreato-Biliary Surgery, Sheba Medical Center, Tel Hashomer, Israel; 7 Department of Pathology, Sheba Medical Center, Tel Hashomer, Israel; University of Minnesota, United States of America

## Abstract

**Purpose:**

To develop a diffusion-tensor-imaging (DTI) protocol that is sensitive to the complex diffusion and perfusion properties of the healthy and malignant pancreas tissues.

**Materials and Methods:**

Twenty-eight healthy volunteers and nine patients with pancreatic-ductal-adenocacinoma (PDAC), were scanned at 3T with T2-weighted and DTI sequences. Healthy volunteers were also scanned with multi-b diffusion-weighted-imaging (DWI), whereas a standard clinical protocol complemented the PDAC patients’ scans. Image processing at pixel resolution yielded parametric maps of three directional diffusion coefficients λ1, λ2, λ3, apparent diffusion coefficient (ADC), and fractional anisotropy (FA), as well as a λ1-vector map, and a main diffusion-direction map.

**Results:**

DTI measurements of healthy pancreatic tissue at b-values 0,500 s/mm^2^yielded: λ1 = (2.65±0.35)×10^−3^, λ2 = (1.87±0.22)×10^−3^, λ3 = (1.20±0.18)×10^−3^, ADC = (1.91±0.22)×10^−3^ (all in mm^2^/s units) and FA = 0.38±0.06. Using b-values of 100,500 s/mm^2^ led to a significant reduction in λ1, λ2, λ3 and ADC (p<.0001) and a significant increase (p<0.0001) in FA. The reduction in the diffusion coefficients suggested a contribution of a fast intra-voxel-incoherent-motion (IVIM) component at b≤100 s/mm^2^, which was confirmed by the multi-b DWI results. In PDACs, λ1, λ2, λ3 and ADC in both 0,500 s/mm^2^ and 100,500 s/mm^2^ b-values sets, as well as the reduction in these diffusion coefficients between the two sets, were significantly lower in comparison to the distal normal pancreatic tissue, suggesting higher cellularity and diminution of the fast-IVIM component in the cancer tissue.

**Conclusion:**

DTI using two reference b-values 0 and 100 s/mm^2^ enabled characterization of the water diffusion and anisotropy of the healthy pancreas, taking into account a contribution of IVIM. The reduction in the diffusion coefficients of PDAC, as compared to normal pancreatic tissue, and the smaller change in these coefficients in PDAC when the reference b-value was modified from 0 to 100 s/mm^2^, helped identifying the presence of malignancy.

## Introduction

MR-Imaging of the pancreas has been increasingly adopted in recent years as a useful tool for the diagnosis and management of a vast variety of pathological conditions, including congenital deformations, inflammatory disease and different origins of neoplasms [1]. However, in spite of MRI advances, evaluation of solid lesions in the pancreas has remained a radiological challenge; mainly in the discrimination between non-neoplastic conditions such as focal pancreatitis and the most common and concerning neoplasm of the pancreas, pancreatic ductal adenocarcinoma (PDAC) [2]. Unfortunately, PDAC carries a poor prognosis and the five year survival rate is below 5% when combined for all stages [3]. Early detection followed by surgical resection offers a hope for cure, however, PDAC usually manifests with clinical symptoms only in advanced unresectable disease. Recently, an important study, illustrating the genetic evolution of PDAC, demonstrated that distant metastasis occurs late during the genetic evolution of pancreatic cancer, suggesting a sufficient window for early detection [4]. This demonstrates the unmet need for improved screening and diagnostic tools in order to identify pancreatic cancer while the tumor is still localized and amenable to surgical resection.

Several MRI methodologies have been applied in pancreatic clinical studies. Magnetic resonance cholangiopancreatography (MRCP) has been shown to be an accurate mean for detecting pancreatic ductal obstruction and evaluating the level and causes of obstruction [5]. Recently quantitative analysis of dynamic contrast enhanced MRI has been applied for evaluating pancreatic tumors, showing a significant correlation between histo-pathological and model based physiological parameters [6]. With the advancement in abdominal MRI diffusion weighted imaging (DWI) protocols have been investigated as a complementary tool for detecting PDAC. However, variable values of the apparent diffusion coefficient (ADC) of PDAC have been reported, with lower and higher ADC values of PDAC as compared with normal pancreatic tissue [7]–[16]. These conflicting results were most likely due to the application of different DWI experimental protocols and processing means. Extended diffusion studies, including diffusion protocols with multi b-values that allowed fitting to a bi-exponential decay, indicated a fast diffusion component at low b-values. This component was attributed to a pseudo diffusion intra-voxel incoherent motion (IVIM), generated by the blood flow in the tortuous microcirculation of the normal pancreatic tissue [14]–[19]. Furthermore, multi-b DWI studies of PDAC indicated that the fraction of the IVIM diffusivity, was a superior parameter for differentiating PDAC from healthy tissue as compared to ADC values [14], [15], [17]–[19].

In general, DWI experiments yield an average ADC over three orthogonal directions, disregarding the anisotropy of the diffusion process in the structured tissue. Alternatively, diffusion tensor imaging (DTI) measures the diffusion coefficients in well-defined directions and extends the ability of DWI to reveal diffusion anisotropy, thereby providing information related to tissue microstructural features [20]. Indeed, DTI was shown to provide significant characterization of tissue microstructure and pathophysiology, originally of the central nervous system, and later of the peripheral nervous system [21]–[23]. DTI was also applied to investigate the architecture remodeling in the myocardium after infarction [24], the diffusion in normal prostate and prostate cancer [25], [26], in normal and pathological kidneys [27], [28], and in normal and pathological liver [29], [30]. More recently it was applied in the breast, showing hormonal regulation among healthy volunteers and its ability to diagnose breast cancer [31], [32]. Thus far the potential of DTI to reveal the complex microstructure and physiology of the pancreas and detect pathological changes have not been investigated.

The pancreas consists of lobules in the range of 2 mm in diameter [33], comprising both endocrine and exocrine elements. Volume-wise, the exocrine element, termed the lobular duct, is the predominant structural component in the normal pancreas and is composed of acini and a ductal system [33]. The endocrine element appears in the form of islets of Langerhans, scattered in each lobule and accounts for less than 2% of each lobule volume [34]. However, within each lobule the islet capillary glomerular network dominates the blood supply and shows approximately five times increased density, as well as more tortuous and permeable capillaries in comparison to the capillary network of the acini [35]. Based on these structural and physiological features and the ability of DTI to track the microstructure of brain fibers [36] and breast ductal system [37] we predicted that DTI of the normal pancreas, using a voxel size close to that of a lobule size, will be primarily affected by the exocrine microstructure and by the endocrine microvascular physiology. Herein, we describe a pilot study of abdominal DTI, focusing on evaluating the feasibility of DTI protocols complemented by a multi-b DWI protocol to characterize the origin and quantify the values of the diffusion tensor parameters of the normal pancreas. Furthermore, we present a preliminary clinical study of pancreatic cancer applying a distinct protocol that enabled identifying significant changes in the DTI measurements of PDAC as compared to normal pancreatic tissue.

## Materials and Methods

### Volunteers

All protocols were approved by the Internal Review Board of Sheba Medical Center, Tel-Hashomer, Israel and a signed informed consent was obtained from all volunteers. From February 2012 to March 2013, 28 healthy volunteers, including 12 females (mean age 39.8, range 22 to 70) and 16 males (mean age 40.0, range 25 to 69), have participated in this prospective study. During this period, five volunteers were scanned twice for repeatability evaluation. All volunteers were in good health without any significant medical history, including diabetes or any other pancreatic diseases.

Additionally, From December 2013 to July 2014, nine patients with PDAC, including one female and eight males (mean age 63.6, range 55 to 78) were scanned. All PDACs were confirmed by biopsy (mean tumor size 2.0±0.7 cm). Five PDACs were located in the pancreas head, three in the body and one in the tail.

### MRI Protocols

All volunteers fasted at least 4 hours before the MRI examination. The MRI protocols were acquired on a 3 Tesla whole body MRI scanner: MAGNETOM Trio, Tim System (Siemens, Erlangen, Germany) equipped with a transmitting body coil and a receiving, multi-channels, body matrix and spine matrix coils (Siemens, Erlangen, Germany). A bellows belt (Siemens, Erlangen, Germany) was placed on the chest for respiratory triggering, and a dielectric pad (Siemens, Erlangen, Germany) placed on the abdomen was applied in order to avoid effects due to radio-frequency interference [38].

The DTI protocol was acquired with fat-suppressed, respiratory triggered twice refocused spin-echo sequence [39], using 30 diffusion gradients directions at b-values of 0, 500 s/mm^2^ (n = 10) and b-values 0, 100 and 500 s/mm^2^ (n = 18), echo time/repetition time (TE/TR) of 75/6000 ms, generalized auto-calibrating partially parallel acquisition (GRAPPA) with parallel imaging factor 2, and spatial resolution of 2×2×2.5 mm^3^ or 3×3×4 mm^3^. The nominal acquisition-time of the DTI 0, 500 s/mm^2^ scans was 3∶24 min and of b = 0,100, 500 6: 18 min.

A multi-b DWI protocol was added after the DTI protocol for the last 12 volunteers. It was applied using fat-suppression, respiratory triggered twice refocused spin-echo sequence, in 3 orthogonal diffusion gradients, at nine b-values: 0, 20, 40, 70, 100, 250, 400, 600 and 800 s/mm^2^, TE/TR of 86/6000 ms, GRAPPA with parallel imaging factor 2, and at the same spatial resolution as the DTI protocol. The final nominal acquisition-time was 2∶48 min.

All examinations included a T2–weighted axial fast 2-dimensional protocols, without and with fat suppression, using a respiratory triggered spin-echo sequence with TE/TR of 59/3000 ms, GRAPPA with parallel imaging factor 2 and the same slice thickness as in the DTI protocol. The nominal acquisition time was 2∶52 min.

PDAC patients (n = 9), were scanned in addition to the above T2–weighted and DTI protocols with a standard pancreas MRI clinical protocol [40] that included axial in-phase and out of phase T1-weighted images with breath holding, coronal 2D and 3D thick-slab MR-cholagio-pancreatography (MRCP), and T1-weighted dynamic contrast enhanced (DCE) imaging with breath holding, before and after automatic injection of Gadopentetate dimeglumine (Magnetol 0.5M, Soreq, Israel). Additional imaging modalities including PET-CT were used for further radiological workup in some of the PDAC patients, but were not quantitatively assessed in the scope of this study.

### Image processing

The diffusion tensor parameters were calculated using a home built software program. This program fits the diffusion coefficient per voxel in each direction according to the Stejskal-Tanner equation [41]. Then it applies a non-linear least square fitting of the resulting diffusion coefficients in each direction to a symmetric tensor yielding six tensor parameters [22]. Application of principal component analysis provides for each voxel three eigenvector (ν_1_, ν_2_, ν_3_) defining the diffusion direction in three orthogonal axes coinciding with the diffusion frame of the tissue. The corresponding three diffusion eigenvalues determine three directional diffusion coefficients arranged from high to low values λ_1_, λ_2_, λ_3_. The average of these three diffusion coefficients yields the apparent diffusion coefficient (ADC) and their values allow calculation of the fractional anisotropy (FA) index as previously described [22]. The diffusion tensor parameters were separately calculated for the pair of b-values 0, 500 s/mm^2^ and for the pair of b-values 100,500 s/mm^2^, using for b = 100 s/mm^2^ the signal intensity over 30 directions averaged to a single scan intensity.

Average values of the DTI parameters in healthy volunteers were calculated in pancreatic regions of interest (ROIs) that were manually delineated on the b = 0 images with the aid of the T2-weighted images, and were then transferred automatically to the parametric maps (see S1 Fig.). Localization of the pancreas regions included the head ROI, right to the left border of the superior mesenteric vein, body ROI, left to the superior mesenteric vein towards the left border of aorta and tail ROI localized in the region left to the left border of the aorta.

Additionally, for validating the DTI scanning protocol and image processing tool we analyzed DTI datasets of the kidney. ROIs of the right kidney and the cortex and medulla were manually delineated on the b = 0 images and transferred automatically to the DTI parametric maps.

Analysis of the multi-b DWI datasets was performed on the mean signal intensity of a pancreatic ROI manually delineated on the b = 0 images. The normalized diffusion intensity S_b_/S_0_ as a function of the b-value was fitted to a bi-exponential decay as previously described [14], [16], [42]:
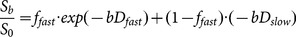
(1)Where *f*_fast_ and *D_fast_* are the fraction and diffusion coefficient respectively of a fast diffusion component and *D_slow_* is the diffusion coefficient of a second slower diffusion process. The fitting of the normalized signal intensities as a function of b-values and the assessment of the goodness of fitting (R-square) were performed using the Trust-Region algorithm which is based on the Levenberg-Marquardt algorithm. (Matlab R2011b, Massachusetts, U.S.A). In order to avoid convergence to a false local minimum and obtain stable results, *D_fast_* and *f_fast_* were applied as free parameters and *D_slow_* was limited to the range: 1.1–1.7 mm^2^/s, based upon our prior results using DTI with b-values of 100, 500 s/mm^2^.

The ROIs of the PDACs were manually delineated by two expert radiologists in the field of body MRI (S.A. and Y.I.) on T2-weighted, T1-weighted and contrast enhanced images. The ROIs were then reproduced on the respective DTI images, allowed by the protocols’ matched slice thickness, field of view and positioning. Additionally, ROIs of normal pancreas regions, distal to the tumor, were delineated for intra-subject comparison. DTI parameters of both PDAC and normal tissue were separately calculated for the two pairs of b-values 0, 500 s/mm^2^ and 100, 500 s/mm^2^, as described above for the healthy volunteers.

### Statistical analysis

Normality of the distribution of the DTI parameters was tested using Shapiro-Wilk test. Two-way analysis of variance (ANOVA) without replication, followed by post-hoc Tukey’s Honestly Significant Difference (HSD) test were used in order to compare between the diffusion coefficients in the three pancreatic regions. The differences between the males and females DTI parameters were evaluated by unpaired two tailed Student’s t-test. The comparisons between the DTI parameters obtained from the two reference b-values (0 and 100 s/mm^2^) in healthy volunteers and between the DTI parameters obtained for repeatability evaluation were evaluated by paired two tailed Student’s t-test. The differences between PDAC and distal normal pancreatic tissue in the same patients were also evaluated by paired two tailed Student’s t-test. The repeatability was also evaluated by intra-class correlation (ICC) (STATISTICA 12.0, StatSoft). Statistical significance was defined as *p*<0.05.

## Results

### DTI of the pancreas in healthy volunteers

**Figure 1:**
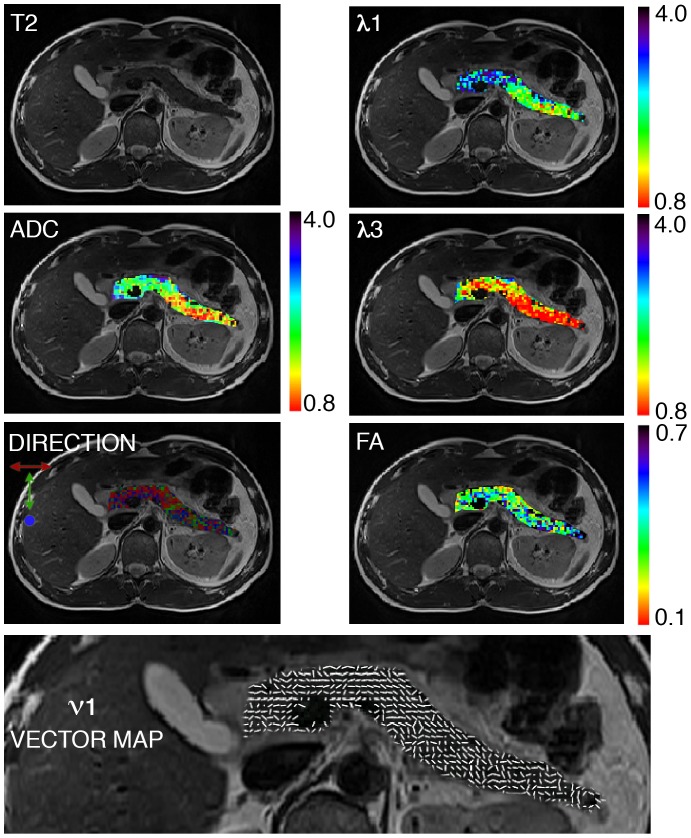
Diffusion tensor parametric and vector maps throughout the entire pancreas of a healthy male volunteer (age 38). Maps are overlaid on the corresponding T2 weighted image. The spatial resolution of the parametric maps is 2×2×2.5 mm^3^. The diffusion coefficients λ1, λ_3_, ADC and FA are defined in Materials and Methods and were analyzed using b = 0, 500 s/mm^2^. λ_1_, λ_3_, ADC are in units of 10^−3^ (mm^2^/s). The DIRECTION map presents in three colors the direction of the 1^st^ principal eigenvector; red: left to right direction; green: head to feet direction and blue: anterior to posterior direction. VECTOR MAP presents in white sticks the direction of the 1^st^ principal eigenvector ν_1_.

Image processing of the DTI datasets yielded parametric maps of the directional diffusion coefficients, λ_1_, λ_2_, λ_3_, ADC, and FA, as well as a vector map of the prime eigenvector, ν_1,_ and a color coded direction map of this eigenvector. Fig. 1 demonstrates typical normal pancreatic parametric maps of λ_1_, λ_3_, ADC and FA overlaid on a T2 weighted image, calculated for a b-value pair of 0, 500 s/mm^2^. The values of each diffusion coefficient span a similar range, but show a mild gradual decline going from the head to the tail. The direction map of λ_1_ described by three colors indicates a complex structure with the direction along the pancreas main duct being dominant. This direction is also predominant in the vector map of ν_1_ (Fig. 1).

Overall the diffusion tensor parameters obtained for b = 0,500 s/mm^2^ datasets exhibited a normal distribution, yielding the following values for the mean ± standard deviation of λ1 = (2.65±0.35)×10^−3^ mm^2^/s, λ2 = (1.87±0.22)×10^−3^ mm^2^/s, λ3 = (1.20±0.18)×10^−3^ mm^2^/s, ADC = (1.91±0.22)×10^−3^ mm^2^/s, and FA = 0.38±0.06 (n = 28) (Table 1). The parameters converged to values that exhibited an inter-subjects coefficient of variation ranging between 0.12 to 0.16 (Table 1). Further analysis of the DTI parameters in the three pancreatic regions confirmed a decline in the directional diffusion coefficients and ADC from the head through the body and into the tail (Table 1, S2 Fig.). Statistical analysis indicated a significant decline of these coefficients in the tail as compared to the head and body with no significant difference between the head and body and between the FA values in all regions (Table 1).

**Table 1:** DTI parameters in the entire pancreas and in the head, body and tail regions.

	λ1	λ2	λ3	ADC	FA
	2.65±0.35	1.87±0.22	1.20±0.18	1.91±0.22	0.38±0.06
**CV**	0.13	0.12	0.15	0.12	0.16
**Head**	2.82±0.47†	2.01±0.27†	1.30±0.20†	2.04±0.28†	0.37±0.06
**Body**	2.66±0.37	1.88±0.25*	1.21±0.20*	1.92±0.26*	0.38±0.06
**Tail**	2.46±0.28	1.72±0.20	1.09±0.20	1.76±0.20	0.39±0.06

Mean values ± SD of 28 volunteers. λ1, λ2, λ3, ADC and FA are defined in Materials and Methods and were analyzed using b = 0, 500 s/mm^2^. CV – coefficient of variation. λ1, λ2, λ3 and ADC are in units of 10^−3^ (mm^2^/s). †*p* value <0.01 and *p value <0.05 as compared to the pancreas tail. The parameters of the head and body were not statistically different *p* = 0.24–0.35 (ANOVA followed by Tukey’s HSD test).

Comparison between the diffusion parameters of the female (n = 12) and male (n = 16) volunteers, both of a similar age group, showed no significant difference in all DTI parameters (*p* values = ranged between 0.08–0.45). Comparison of the datasets of volunteers that were scanned twice under the same conditions indicated effective repeatability showing no significant difference in the values of the directional diffusion parameters with *p* (λ1, λ2, λ3) = 0.73, 0.93, 0.69 and ICC (λ1, λ2, λ3) = 0.89, 0.86, 0.33.

The validation of the methodology of DTI through the analysis of the kidney DTI parameters yielded the diffusion tensor parameters of the two main regions, the cortex and the medulla as detailed in S1 Table.

**Figure 2:**
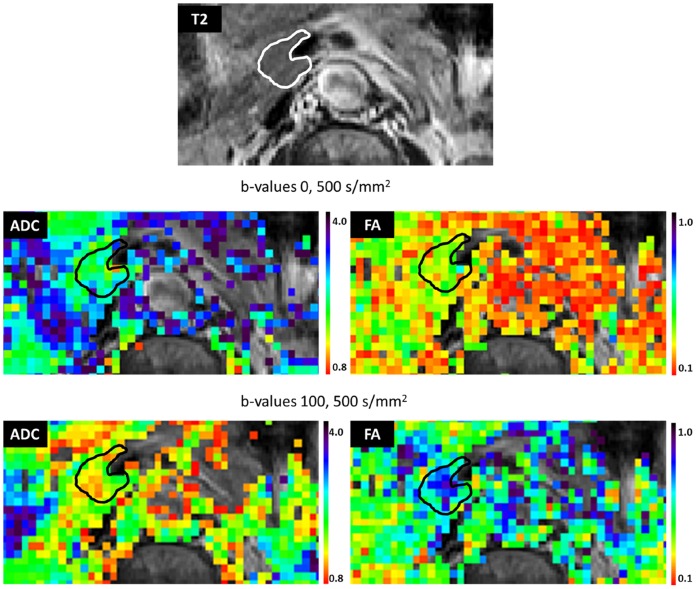
ADC and FA maps in the region of a pancreas head of a healthy female volunteer (age 69), using b-values 0,500 s/mm^2^ and 100, 500 s/mm^2^. Maps are overlaid on the corresponding T2 weighted image. The spatial resolution of the parametric maps is 2×2×2.5 mm^3^. ADC and FA are defined in Materials and Methods. ADC is in units of 10^−3^ (mm^2^/s). Note a decrease in ADC values and an increase in FA values in the 100, 500 s/mm^2^ as compared with the 0,500 s/mm^2^.

**Figure 3:**
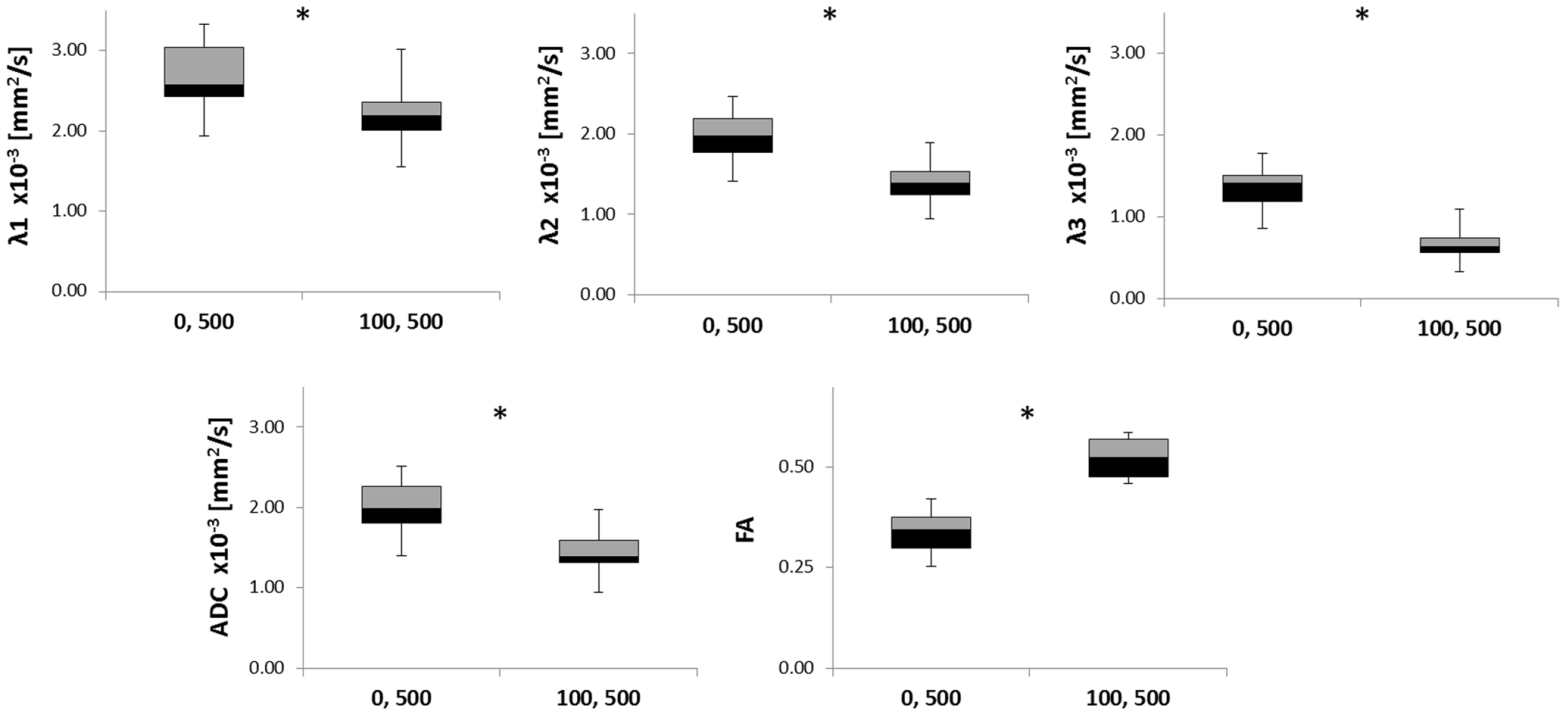
The diffusion tensor measurements of the pancreas using b-values 0, 500 s/mm^2^ and 100, 500 s/mm^2^. Results are demonstrated in box (median ±interquartile range [IQR]) and whiskers (±1.5 IQR) plots (n = 18 healthy volunteers). λ_1_, λ_2_, λ_3_ & ADC are in units of 10^−3^ (mm^2^/s). The figure also includes the mean change in the parameters and the statistical evaluation of the difference between the two sets of parameters obtained by a two tailed student’s t-test. * *p*-value <0.0001.

Further characterization of the diffusion characteristics of the pancreas was performed by comparing the DTI parameters of the same volunteer using b-values = 0, 500 s/mm^2^ and b values 100, 500 s/mm^2^. For the b = 100,500 s/mm^2^ the mean ± standard deviation were: λ1 = (2.15±0.43)×10^−3^ mm^2^/s, λ2 = (1.34±0.26)×10^−3^ mm^2^/s, λ3 = (0.66±0.20)×10^−3^ mm^2^/s, ADC = (1.38±0.27)×10^−3^ mm^2^/s, and FA = 0.51±0.07 (n = 18) (Figs. 2 and 3, S2 Table). The comparison between these two sets of results (Figs. 2 and 3) indicated a significant a reduction of 18% to 50% (*p*<.0001, paired Student’s t-test) in the directional diffusion coefficients and ADC due to the change in the reference b-value from 0 to 100 s/mm^2^. However, this change led to a significant increase (*p*<.0001, paired Student’s t-test) in FA (Figs. 2 and 3, S2 Table). The significant decline in the diffusion coefficients suggested the presence of a fast diffusion component dominating the signal decay at b≤100 s/mm^2^ which was excluded when a reference b-value of 100 s/mm^2^ was used.

### DWI of the pancreas in healthy volunteers

**Figure 4:**
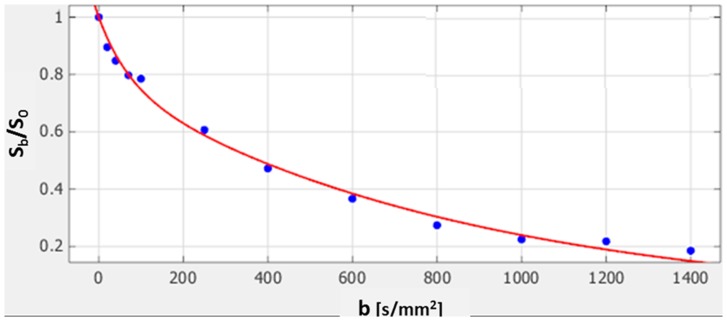
The decay of the normalized signal intensity in a multi b-value DWI experiment of the pancreas. The mean values in ROI of a pancreas head of a healthy male volunteer (age 65). The best fitted bi-exponential decay curve (in red) yielded a fast decay component *D_fast_* of 48.96×10^−3^ mm^2^/s with a fraction of this component *f*_fast_ of 12.18% and a slow diffusivity *D_slow_* of 1.44×10^−3^ mm^2^/s. R-square was 0.99 assessing the goodness of the fitting as described in Materials and Methods.

Further exploration of the presence of the fast diffusion component in the pancreas was performed by scanning volunteers with a multi b-value DWI protocol. Analysis of the DWI datasets indicated a fast decay component at low b-values, with a reduction in the signal intensity of (29±5) % (range: 22–40%) between b = 0 to b = 100 s/mm^2^, followed by a second component with a slower decay. Bi-exponential fitting of the decays according to equation 1 (Fig. 4) yielded the values of the fast and slow diffusion components, *D_fast_* and *D_slow_,* respectively, and of the fast component fraction, *f*_fast_ (Table 2). The diffusion coefficient of the fast component *D_fast_* was very high [(56.95±22.84)×10^−3^ mm^2^/s] with a relatively high fraction of 30.04±13.10. The slow diffusion coefficient, *D_slow_*, was relatively low [(1.22±0.25)×10^−3^ mm^2^/s] and close to that obtained for ADC from DTI with a reference b-value of 100 s/mm^2^ (*p* = 0.41).

**Table 2:** Diffusion parameters derived from multi-b DWI experiments of the pancreas.

	*D_slow_*	*D_fast_*	*f_fast_ (%)*	*R-square*
	x10^−3^ mm^2^/s	*x10* ^−*3*^ * mm^2^/s*		
**Mean±SD**	1.22±0.25	56.95±22.84	30.04±13.10	0.97±0.20
**Range**	[1.10–1.70]	[20.00–92.92]	[10.31–46.49]	[0.95–0.99]

Mean values ± SD of 12 volunteers. The diffusion coefficients, *D_slow_, D_fast_,* and the fraction, *f_fast_*, as well as the fitting to a bi-exponential decay (Equation 1) are described in Materials and Methods.

### DTI measurements of patients with PDAC

**Figure 5:**
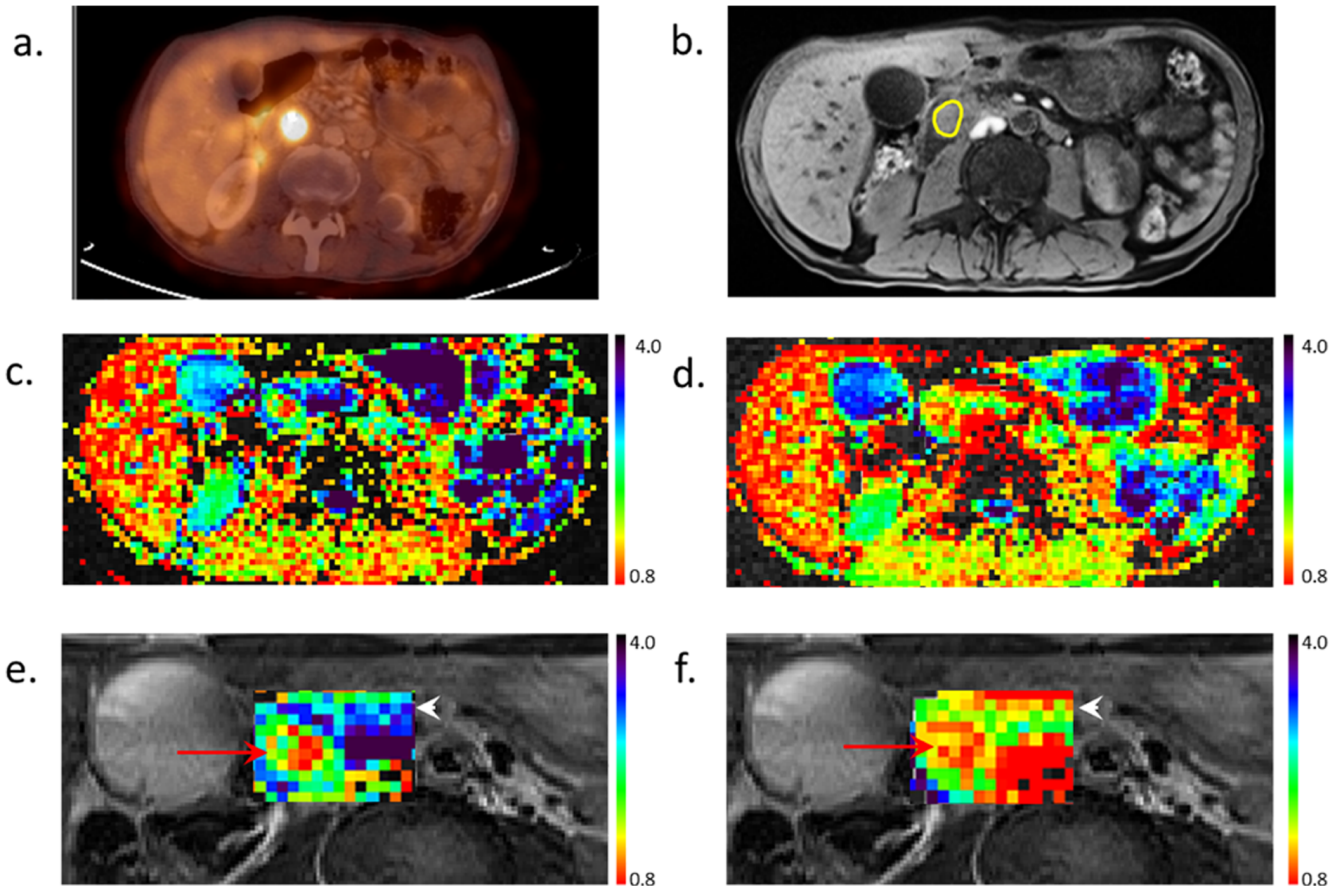
PET-CT, DCE-MRI and ADC maps using b-values 0,500 s/mm^2^ and 100, 500 s/mm^2^ in the region of PDAC of a male patient (age 66). (a) PET-CT exhibiting increased uptake of ^18^F labeled deoxy-glucose (FDG) in the region of pancreatic head (b) T1-weighted, pre-contrast image revealing PDAC location, marked by yellow circle. (c and d) ADC maps of the entire abdominal slice using b-values of 0, 500 s/mm^2^ and 100, 500 s/mm^2^, respectively. (d and e) Zoomed (x2) ADC maps of the regions in c and d that include PDAC and its surrounding, using b-values of 0, 500 s/mm^2^ and 100, 500 s/mm^2^, respectively, overlaid on the corresponding T2-weighted image. *Notice* that the tumor in e (red arrow) is clearly visible on the 0, 500 s/mm^2^ ADC map showing a high contrast with the surrounding normal tissue (white arrow head). However, in f, due to the reduction in ADC of the normal at 100, 500 s/mm^2^ (from 2.22±0.64 to 1.49±0.35 mm^2^/s) and the similarity in the ADC of the tumor (from 1.38±0.37 to 1.24±0.28 mm^2^/s) the contrast between the tumor and the surrounding pancreatic tissue is diminished.

All nine PDACs were identified on the various DTI derived maps, in accordance with their location in T1- weighted, T2-weighted and contrast enhanced images. The mean ± standard deviation of the diffusion coefficients in the ROI of the PDACs using a null b-value as a reference were: λ1 = (1.9±0.3)×10^−3^ mm^2^/s, λ2 = (1.3±0.2)×10^−3^ mm^2^/s, λ3 = (0.8±0.2)×10^−3^ mm^2^/s and ADC = (1.3±0.2)×10^−3^ mm^2^/s, (n = 9; Table 3). These values were found to be significantly lower (p<0.001, paired two tail t-test) than the values of the corresponding diffusion coefficients in the distal normal pancreatic tissue of the patients (Table 3). A significant reduction in the diffusion coefficients of PDACs in comparison to the distal normal tissue was also obtained with the reference b-value of 100 s/mm^2^ (Table 3). In addition, the percent decrease of the various diffusion coefficients between the two reference b-values in PDACs was significantly lower than the corresponding decrease in the distal pancreatic tissue (Table 3). This is further demonstrated in Fig. 5; A representative case of a 66 years old male who was diagnosed with PDAC, identified by standard MRI protocol and FDG increased uptake on PET-CT. The PDAC is clearly visualized with low ADC values at the two reference b-values. While the lesion’s ADC values hardly changed, the ADC of the surrounding normal pancreatic tissue reduced significantly when 100 s/mm^2^ was the reference b-value. These results suggest a smaller contribution of a fast diffusion component, presumably due to IVIM, in the cancer tissue as compared to the normal pancreatic tissue.

**Table 3:** DTI measurements of PDAC patients.

PDAC						
	λ1	λ2	λ3	ADC	FA	
b-values 0,500	1.91±0.27†	1.31±0.15†	0.81±0.15†	1.35±0.18†	0.41±0.06†	
b-values 100,500	2.01±0.30*	1.15±0.20^#^	0.48±0.18	1.22±0.18*	0.56±0.10	
	5.1±7.4†	−12.2±13.5*	−40.2±15.4*	−9.4±10.2†	36.0±13.0†	

Normal distal pancreatic tissue					
	λ1	λ2	λ3	ADC	FA
b-values 0,500	2.69±0.17	1.98±0.16	1.37±0.18	2.01±0.16	0.34±0.04
b-values 100,500	2.47±0.17	1.46±0.19	0.63±0.26	1.52±0.19	0.56±0.08
Change (%)	−8.0±6.7	−25.8±8.0	−54.5±13.1	−24.4±7.5	63.6±22.2

Mean values ± SD of λ1, λ2, λ3, ADC and FA in nine PDAC patients. The results were obtained by analyzing ROIs of PDAC and pancreatic tissue distal to the tumor that appeared normal, using b = 0,500 s/mm^2^ and b = 100,500 s/mm^2^. λ_1_, λ_2_, λ_3_ & ADC are in units of ×10^−3^ (mm^2^/s). The difference in the various DTI measurements between PDAC and normal distal tissue were evaluated by two-ways paired t-test using for statistical significance the following symbols (†)*p*<0.001, (*) *p*<0.01, (^#^)p<0.05.

Moreover, the analysis of DTI datasets indicated that the fractional anisotropy in the ROIs of PDACs was slightly but significantly higher as compared to the distal normal pancreas when a null reference b-value was used, but did not change significantly for a reference b-value of 100 s/mm^2^ (Table 3).

## Discussion

This study demonstrates the feasibility of diffusion tensor imaging of the normal pancreas to yield consistent parametric maps of the directional diffusion coefficients coinciding with the diffusion frame of the tissue and the diffusion fractional anisotropy. Since the voxel size in our study was of the order of a normal pancreatic lobule size [33], the diffusion tensor parameters per voxel primarily reflect the lobule ductal-acini microstructure and may include a contribution from the pseudo diffusion due to micro-capillary tortuous flow in each lobule. As this study is the first to measure the diffusion tensor parameters of the pancreas we could only compare the ADC values obtained in our study to those obtained previously by DWI experiments. The mean ADC values determined from the DTI analysis with a reference null b-value were similar to values obtained earlier by DWI studies which used equal or similar b-values [7]–[9], [12], [14], [43]. Lower ADC values were obtained with protocols that applied higher b-values (up to b = 1000 s/mm^2^) [10], [11]. In addition to the lower SNR at high b values this can be explained by the fact that at high b-values when the fast decay of the extracellular signal is almost completed, the intracellular contribution of restricted diffusion becomes dominant, leading to a slow-down of the decay, as was shown previously *in* *vivo* [44]. Analysis with a uni-exponential equation, using a reference b-value and a high b-value (such as 1000 s/mm^2^) yields a reduced apparent diffusion coefficient as compared to a similar analysis with a significantly lower b-value (such as b = 500 s/mm^2^) which is dominated by the extracellular contribution. Thus, for a uni-exponential decay model, ADC reduction at high b-values as compared to low b-values mainly reflects the higher contribution of the restricted intracellular diffusion to the overall diffusion process in the tissue. Lower values of ADC were also obtained for a reference b≥50 s/mm^2^ [13], [45], [46] and were similar to the values we obtained with initial b = 100 s/mm^2^. This is expected when there is a fast diffusion component in addition to the extracellular diffusion, and a uni-exponential model is used.

To further validate our DTI methodology we compared our results with the results of previously published kidney DTI studies [27], [47]–[52]. The values of ADC and FA in the kidney cortex and medulla regions that we have obtained were within the range found previously for these parameters using similar b-values [27], [49], [50]. Our results also showed that the values of λ2, λ3, FA and ADC in the cortex exceeded the medullary values, while λ1 values were similar in both regions, as was previously reported in studies that used also different b-values [27], [48]. This comparison together with the demonstration of good repeatability of our study verified the ability of our abdominal DTI protocol and processing means to determine reliable diffusion tensor parameters.

We further found that depending on the choice of a reference b-value (0 or 100 s/mm^2^), the tensor diffusion coefficients included or excluded a contribution from a fast diffusion process. This finding is in full agreement with previously multi-b value DWI studies of the pancreas [14], [53]. The slow and fast diffusion coefficients and their fractions in our study were in the range obtained previously for the normal pancreas [14], [16], [53], and the encountered differences appear to stem from the use of different methods of fitting. The fast diffusion component was attributed to a pseudo diffusion IVIM caused by the disordered flow in the blood micro-capillary network [42]. Indeed, it is well established that the pancreas is well vascularized with several supplying arteries and has high islet capillary glomerular network in each pancreatic lobule [35], [54], and therefore, it is reasonable to suggest that the fast diffusion component is dominated by the microcapillary perfusion process. Further support for the presence of IVIM in the pancreas was obtained by Lemke et al. using a blood suppression sequence that emphasized the capillary perfusion effect [16]. Nevertheless, the origin of this fast component is still under debate since the fraction of the fast diffusion component found in the DWI studies is relatively high compared to the actual microcapillary fraction measured by other methods [55]. Resolving this discrepancy, require simultaneous detailed diffusion and perfusion MRI studies and using more complex diffusion models, that also take into account the various tissue compartments (such as extracellular, intracellular and intravascular environments), as well as water exchange between these environments and restriction processes within them [56]–[57].

The characterization of the DTI parameters in the normal pancreatic tissue was followed by investigating these measurements in nine PDACs, searching for independent parameters that will provide sufficient contrast for differentiating pancreatic cancer from normal tissue. Our findings of decreased diffusivity (λ1, λ2, λ3 and ADC) in the PDACs as compared with the distal pancreatic tissue, with both reference b-values were in accordance with previous DWI studies, reporting lower ADC values in pancreatic cancer attributed to their higher cellularity [7]–[10], [12].

We also investigated the change in the DTI measurements in PDACs as compared to the distal pancreatic tissue, when the reference null b-value was modified from 0 to 100 s/mm^2^. The changes in the distal tissue were similar to those obtained in the volunteers with healthy pancreatic tissue (Fig. 5, Table 3 and S2 Table). However, in PDACs, these changes were significantly lower as compared with the distal tissue. Thus, the smaller change in the diffusion coefficients between the two datasets 0, 500 s/mm^2^ and 100, 500 s/mm^2^, may provide an independent indication for differentiating cancerous from normal pancreatic tissue. It is reasonable to suggest that this indication reflects reduced contribution of the IVIM- fast diffusion component, in agreement with the decreased perfusion fraction reported in IVIM studies of PDAC [14]–[15], [17]–[19] and the diminished enhancement of PDACs in the arterial phase of contrast enhancement [40], [58].

The FA of the normal pancreas in healthy volunteers (Fig. 5) and in distal pancreatic regions in PDAC patients (Table 3) significantly increased when the reference null b-value was modified to a reference b-value of 100 s/mm^2^. Similarly, FA significantly increased in PDAC, although this increase was less pronounced than the increase in the normal tissue. This increase could stem from the decrease in ADC due to the elimination of the IVIM contribution since the scaling of FA between 0 to 1 depends on ADC causing FA to increase with decreased ADC for the same absolute differences between the diffusion coefficients. However, in PDACs we cannot exclude an explanation that the increase is due to an actual change in the microstructure anisotropy. Further studies including comparison with histological features and blood vessels immune-staining may clarify this issue.

Generally in this this work, we were confronted by technical limitations which are common to abdominal MRI such as rf interference and motional artifacts due to breathing [59], [60]. Furthermore, the EPI based diffusion protocols had additional limitations due to gradient eddy currents, B_0_ field inhomogeneity and susceptibility differences [61]–[63]. Part of the above limitations were reduced by using a dielectric pad and a bellows belt for respiratory triggering, as well as a spin-echo twice refocused echo-planar-imaging sequence that reduces geometrical distortions. A specific methodological limitation related to the contribution of IVIM is the indirect assessment of this contribution by using three b-values (0, 100 and 500 s/mm^2^) and calculating the change in the diffusion coefficients using two sets of b-values, 0, 500 s/mm^2^ and 100, 500 s/mm^2^. In our DWI study of IVIM in the healthy volunteers and in previous DWI and IVIM studies of the pancreas [14], [16]–[19] this information has been obtained by applying 10 or more b-values. However, such a DWI protocol and the limitations of the biexponential fitting, in particular at pixel resolution, are difficult to perform in a standard setting of a clinical examination of the pancreas. Indeed, in most clinical DWI protocols for ADC measurements two b-values are applied assuming a uniexponential decay reflecting the extracellular diffusion. Clearly the difference between the diffusion coefficients for the two reference b-values cannot determine the exact contribution of the fast-IVIM contribution, but as was shown in the healthy pancreas it can serve to indicate the presence of IVIM and it is sensitive to reduction in this component as was shown in the PDACs. By using the difference in the calculated ADC between the two reference b-values, the exact contribution of IVIM has been compromised but assessment of changes in IVIM contribution could be extracted with straightforward image processing tools and within a reasonable time frame. We specifically chose a relatively low second b-value of 500 s/mm^2^ in order to probe primarily the extracellular diffusion and achieve SNR above 2.0 for maintaining Gaussian noise distribution [64]. Thus, the three b-value protocol and two uniexponential fittings present a practical need that still provides consistent and reproducible ADC values and an indirect assessment of the IVIM contribution, both at pixel resolution.

In this study a small cohort of PDAC patients was examined and therefore the results describing the diffusion in pancreatic cancer should be interpreted with caution.

Further studies of patients with suspicious pancreatic malignancy are underway in order to substantiate the clinical utility of the DTI protocol and continue developing image processing tools for PDAC detection by MRI.

In conclusion, DTI of the pancreas is feasible and appears to yield information compatible with the structural and physiological features of the healthy organ and to detect distinct diffusion features of PDACs. We found that the diffusion coefficients of the healthy pancreatic tissue change depending on the reference b-value, exhibiting a notable contribution from a fast diffusion component when the reference b-value is null, attributed to IVIM. Identifying changes in the diffusion tensor parameters of pancreatic cancers may help improve MRI of the pancreas. Indeed, in this preliminary study of PDAC, we found a significant reduction in the directional diffusion coefficients and a lower contribution of a fast-IVIM component in the cancerous tissue.

## Supporting Information

S1 Fig **T2, b-zero and ADC map in the region of a pancreas head of a healthy male volunteer (age 38), using b-values 0,500 s/mm^2^, demonstrating representative ROI delineation.** ROI of the pancreas is manually delineated on the b = 0 image assisted by the corresponding T2-weighted image, and automatically transferred to the ADC map, as well as to the other parametric maps (not shown).
(TIF)

S2 Fig **DTI parameters in the head, body and tail of normal pancreatic tissue.** The results are demonstrated in box (median ±interquartile range [IQR]) and whiskers (±1.5 IQR) plots (n = 28 healthy volunteers). × indicates minimum or maximum values falling below or above the range, respectively. λ_1_, λ_2_, λ_3_ & ADC are in units of 10^−3^ (mm^2^/s). The T2 image in the right side of the 2^nd^ row presents an example of the ROIs of the three pancreatic regions on an axial slice: H- head, B-body and T-Tail.
(TIF)

S1 Table **DTI parameters in the cortex and medulla of the kidney.**
(DOCX)

S2 Table **The effect of the reference b value (0 or 100** **s/mm^2^) on the diffusion measurement of the healthy pancreas.**
(DOCX)
