# Supplementary figures and images for: Diffusion Tensor Magnetic Resonance Imaging of the Pancreas
Source: PLoS One. 2014 Dec 30;9(12):e115783. doi: 10.1371/journal.pone.0115783 (PMC4280111; doi:10.1371/journal.pone.0115783)

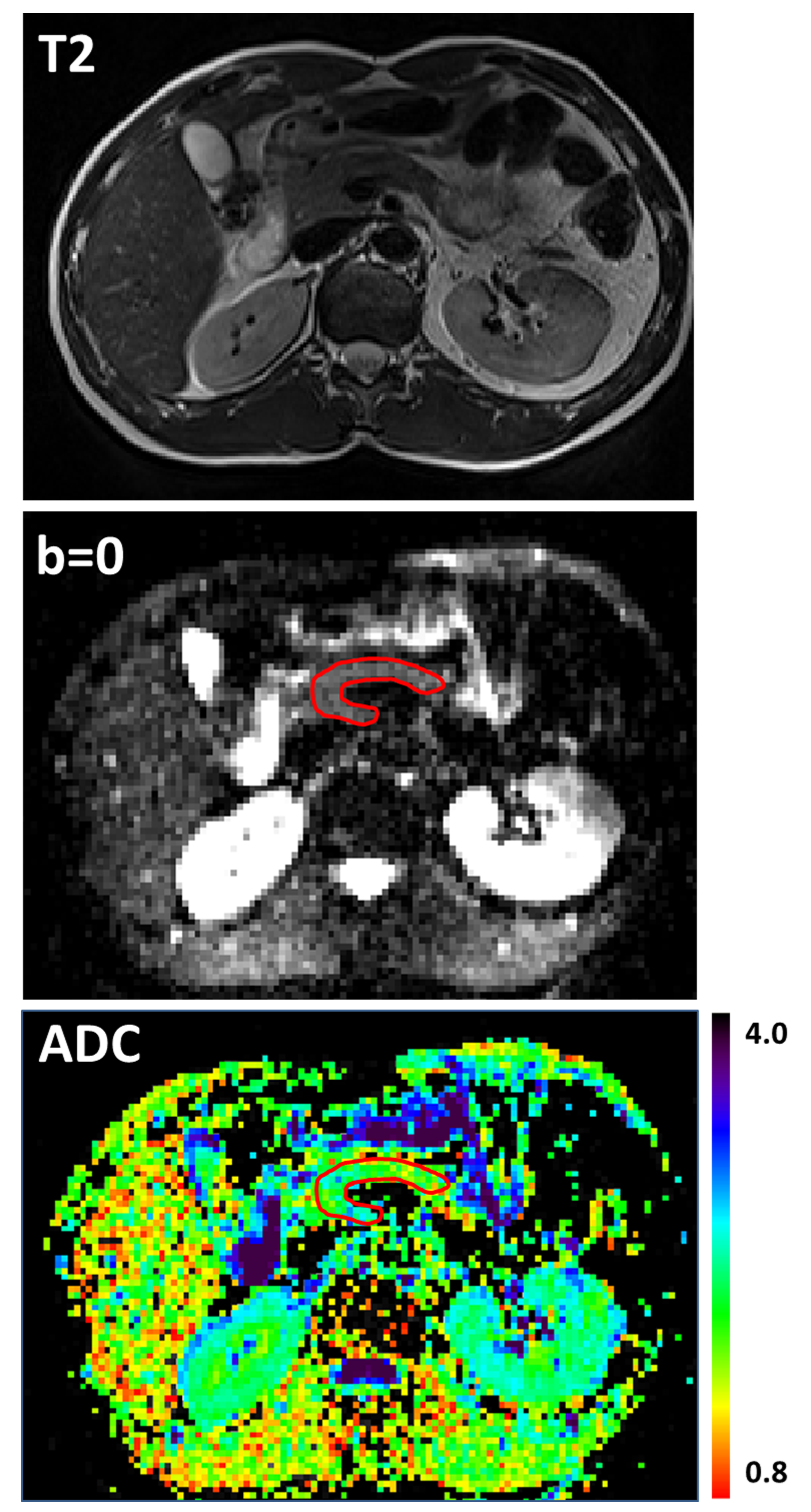

Supplement: S1 Fig — T2, b-zero and ADC map in the region of a pancreas head of a healthy male volunteer (age 38), using b-values 0,500 s/mm2, demonstrating representative ROI delineation. ROI of the pancreas is manually delineated on the b = 0 image assisted by the corresponding T2-weighted image, and automatically transferred to the ADC map, as well as to the other parametric maps (not shown). (TIF) [file pone.0115783.s001.tif]

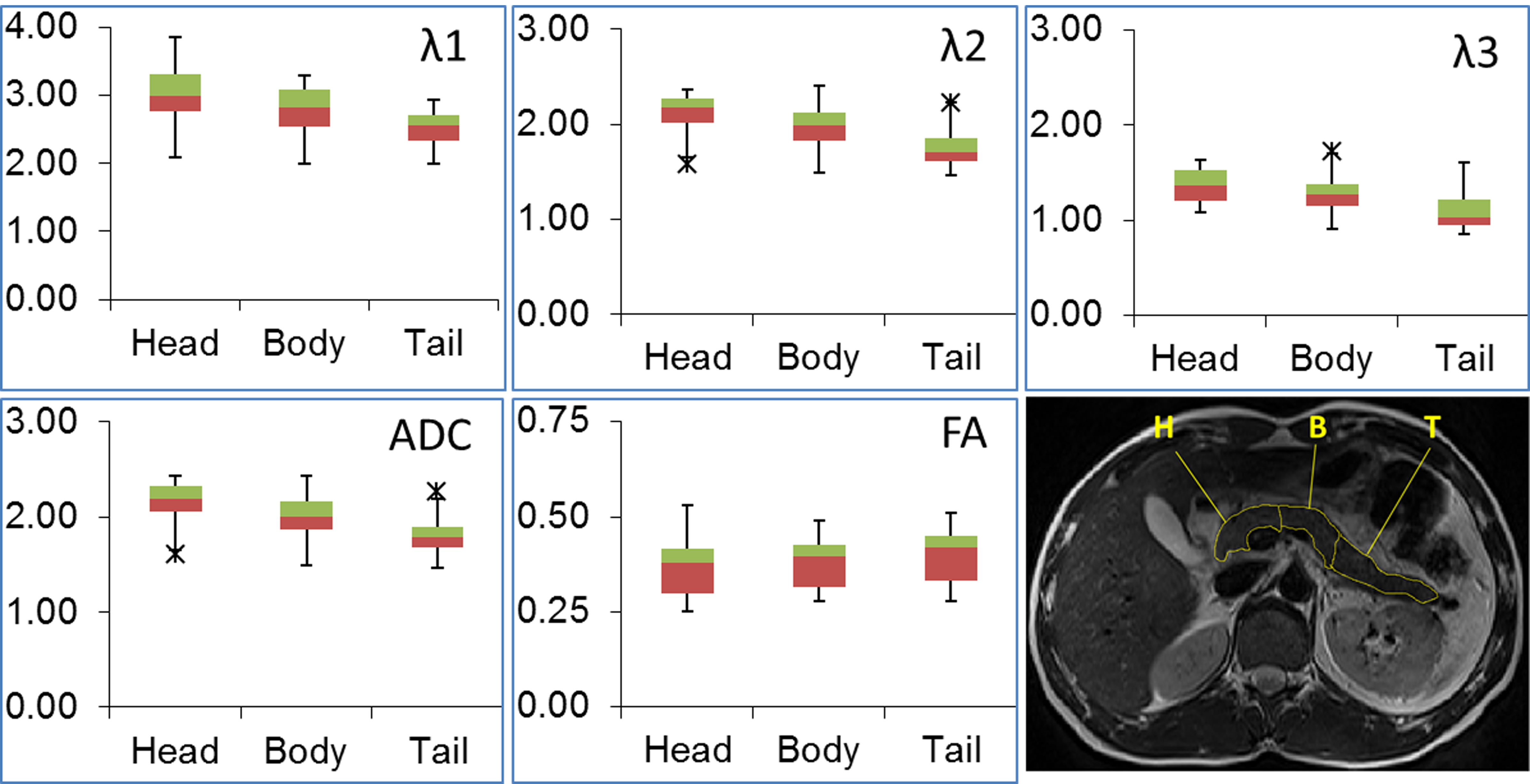

Supplement: S2 Fig — DTI parameters in the head, body and tail of normal pancreatic tissue. The results are demonstrated in box (median ±interquartile range [IQR]) and whiskers (±1.5 IQR) plots (n = 28 healthy volunteers). × indicates minimum or maximum values falling below or above the range, respectively. λ1, λ2, λ3 & ADC are in units of 10−3 (mm2/s). The T2 image in the right side of the 2nd row presents an example of the ROIs of the three pancreatic regions on an axial slice: H- head, B-body and T-Tail. (TIF) [file pone.0115783.s002.tif]
