# Supplementary material for: Diffusion Tensor Magnetic Resonance Imaging of the Pancreas
Source: PLoS One. 2014 Dec 30;9(12):e115783. doi: 10.1371/journal.pone.0115783 (PMC4280111; doi:10.1371/journal.pone.0115783)
Supplement: S1 Table — DTI parameters in the cortex and medulla of the kidney. (DOCX) [file pone.0115783.s003.docx]

**Table S1**

**DTI parameters in the cortex and medulla of the kidney.**

Mean values ± SD of the right kidney in ten volunteers. λ1, λ2, λ3, ADC and FA are

defined in Materials and Methods and were analyzed using b=0, 500 s/mm^2^. λ1, λ2, λ3 and ADC are in units of 10^-3^ (mm^2^/s). *p*-values were obtained by two tailed paired Student’s t-test.

|  | **Cortex** | **Medulla** | ***p* value** |
| --- | --- | --- | --- |
| **λ1** | 3.09 ± 0.22 | 3.07 ± 0.20 | 0.8 |
| **λ2** | 2.38 ± 0.15 | 2.14 ± 0.18 | <0.005 |
| **λ3** | 1.92 ± 0.14 | 1.63 ± 0.17 | <0.001 |
| **FA** | 0.24 ± 0.04 | 0.32 ± 0.05 | <0.005 |
| **ADC** | 2.46 ± 0.14 | 2.28 ± 0.15 | <0.01 |
