# Supplementary material for: Diffusion Tensor Magnetic Resonance Imaging of the Pancreas
Source: PLoS One. 2014 Dec 30;9(12):e115783. doi: 10.1371/journal.pone.0115783 (PMC4280111; doi:10.1371/journal.pone.0115783)
Supplement: S2 Table — The effect of the reference b value (0 or 100 s/mm2) on the diffusion measurement of the healthy pancreas. (DOCX) [file pone.0115783.s004.docx]

**Table S2**

**The effect of the reference b value (0 or 100 s/mm^2^) on the diffusion measurement of the healthy pancreas**

Mean values ± S.D. of 18 volunteers. λ1, λ2, λ3 and ADC are defined in Materials and Methods. λ1, λ2, λ3 and ADC are in units of 10^-3^ (mm^2^/s). * *p*-value <0.0001.

|  | | λ1 | λ2 | λ3 | ADC | FA |
| --- | --- | --- | --- | --- | --- | --- |
| b-values 0,500  b-values 100,500 |  | 2.70 ± 0.30 | 1.98 ± 0.22 | 1.36 ± 0.24 | 2.04 ± 0.28 | 0.33 ± 0.04 |
|  |  | 2.09 ± 0.25* | 1.38 ± 0.13* | 0.84 ± 0.12* | 1.44 ± 0.16* | 0.42 ± 0.06* |
| Change (%) |  | -22.1 ± 9.5 | -29.8 ± 8.7 | -36.0±15.0 | -28.4 ± 9.2 | 24.2 ± 17.5 |

*p* values were obtained by two tailed paired Student’s t-test between datasets of b-values 0,500 as compared with b-values 100,500
